# Predicting the Disease Genes of Multiple Sclerosis Based on Network Representation Learning

**DOI:** 10.3389/fgene.2020.00328

**Published:** 2020-04-21

**Authors:** Haijie Liu, Jiaojiao Guan, He Li, Zhijie Bao, Qingmei Wang, Xun Luo, Hansheng Xue

**Affiliations:** ^1^Department of Neurology, Xuanwu Hospital, Capital Medical University, Beijing, China; ^2^Department of Physical Medicine and Rehabilitation, Tianjin Medical University General Hospital, Tianjin, China; ^3^Stroke Biological Recovery Laboratory, Department of Physical Medicine and Rehabilitation, Spaulding Rehabilitation Hospital, The Teaching Affiliate of Harvard Medical School Charlestown, Boston, MA, United States; ^4^School of Computer Science, Northwestern Polytechnical University, Xi'an, China; ^5^Department of Automation, College of Information Science and Engineering, Tianjin Tianshi College, Tianjin, China; ^6^School of Textile Science and Engineering, Tiangong University, Tianjin, China; ^7^Kerry Rehabilitation Medicine Research Institute, Shenzhen, China; ^8^Shenzhen Dapeng New District Nan'ao People's Hospital, Shenzhen, China

**Keywords:** multiple sclerosis, network embedding, disease gene prediction, PPI network, deep learning

## Abstract

Multiple sclerosis (MS) is an autoimmune disease for which it is difficult to find exact disease-related genes. Effectively identifying disease-related genes would contribute to improving the treatment and diagnosis of multiple sclerosis. Current methods for identifying disease-related genes mainly focus on the hypothesis of guilt-by-association and pay little attention to the global topological information of the whole protein-protein-interaction (PPI) network. Besides, network representation learning (NRL) has attracted a huge amount of attention in the area of network analysis because of its promising performance in node representation and many downstream tasks. In this paper, we try to introduce NRL into the task of disease-related gene prediction and propose a novel framework for identifying the disease-related genes multiple sclerosis. The proposed framework contains three main steps: capturing the topological structure of the PPI network using NRL-based methods, encoding learned features into low-dimensional space using a stacked autoencoder, and training a support vector machine (SVM) classifier to predict disease-related genes. Compared with three state-of-the-art algorithms, our proposed framework shows superior performance on the task of predicting disease-related genes of multiple sclerosis.

## 1. Introduction

Multiple sclerosis (MS) is an autoimmune disease that disrupts the myelin and axons, which leads to inflammatory disorder of the brain and spinal cord (Compston and Coles, 2002), and it is difficult to find exact pathogens and disease-related genes. In recent studies, some of the disease-related genes of multiple sclerosis have been collected and made available, such as in the DisGeNet database (Pinero et al., 2017). However, there are still many unknown MS disease-related genes that need to be discovered. Identifying such genes will effectively contribute to discovering the inner molecular mechanisms of MS as a disease and will help researchers to learn more about MS. Thus, it is essential and of importance to develop a novel algorithm to identify the disease-related genes of MS rapidly and effectively.

Predicting disease-related genes has attracted a huge amount of attention in recent years, and many computational methods have been proposed because of the natural advantages of such methods in terms of time and money saved (Peng et al., 2017, 2019a, 2020a; Ma et al., 2018a; Hu et al., 2019; Xue et al., 2019b). Furthermore, computational methods are effective and precise enough to guide wet experiments (Liu et al., 2019a,b; Peng et al., 2019c). Thus, it is necessary to explore the area of predicting disease-related genes using computational methods. Most of the existing methods for predicting disease-related genes are based on the assumption of the guilt-by-association hypothesis (Peng et al., 2019a). Specifically, genes associated with the same or similar diseases usually have a higher probability of sharing the same topological structure or similar neighbors as others in the gene interaction networks. Thus, based on this guilt-by-association hypothesis, the core of predicting disease-related genes is calculating the distance or similarity between candidate genes and disease-related genes effectively and correctly.

Many approaches have been proposed to measure distance or similarity between gene nodes. The simplest method is direct neighborhood counting (Oti et al., 2006), which mainly counts the number of disease-related genes among their neighborhoods. If the neighbors of gene *g* are associated with multiple sclerosis disease, gene *g* is likely to be a disease-related gene. However, this method overlooks disease-related genes that do not connect with g in the protein-protein-interaction (PPI) network. To solve this problem, several methods are proposed to utilize the shortest path length model to measure the distance between genes (Krauthammer et al., 2004). However, these methods have not achieved satisfying performance, because both the directing neighborhood counting and shortest path length methods only consider the local topological structure of the PPI network instead of the global information of the network topology. Many papers suggest that global topological information would be able to improve the performance of gene node presentation and downstream tasks (Ma et al., 2018b, 2019; Peng et al., 2019b, 2020b; Xue et al., 2019a). Thus, some papers have tried to capture global topological information through random walk with restart (Li and Patra, 2010; Ma et al., 2017; Peng et al., 2018). Borrowing ideas from random walk with restart, we aim, in the current study, to introduce network representation learning (NRL) methods, which represent genes in the network as low-dimensional features, into the task of predicting the disease-related genes of MS.

In this paper, we implement an existing NRL method, termed NRL-based algorithms, for the task of predicting MS disease-related genes and transform non-linear feature vectors into low-dimensional space with a stacked autoencoder. The contributions of this paper can be listed as follows:

- NRL-based algorithms learn global non-linear topological information of the protein-protein-interaction network based on node2vec, DeepWalk, and LINE.
- The deep learning model of a stacked autoencoder is implemented in our proposed framework to extract low-dimensional feature vectors.
- NRL-based algorithms show superior performance in the task of predicting the disease-related genes of MS.

## 2. Methods

**Figure 1:**
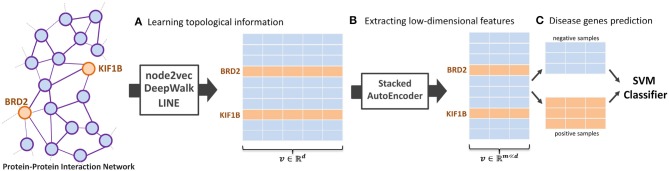
The workflow of the proposed NRL-based framework. The framework contains three main parts: **(A)** learning the topological structure of the protein-protein-interaction network, **(B)** transforming network embedding features into low-dimensional space, and **(C)** training the support vector machine classifier to predict disease-related genes.

In this paper, we introduce NRL algorithms, termed NRL-based algorithms, for the task of predicting the disease-related genes of MS. The framework used contains three main parts: NRL-based algorithms, a Stacked AutoEncoder (Bengio et al., 2006), and a Support Vector Machine (SVM) (Chang and Lin, 2011). Here, we use three classical NRL algorithms to transform the PPI network into high-dimensional feature space, namely node2vec (Grover and Leskovec, 2016), DeepWalk (Perozzi et al., 2014), and LINE (Tang et al., 2015). After obtaining the PPI network embedding features, we run a stacked autoencoder model to extract useful feature vectors into low-dimensional space. Finally, a SVM classifier is implemented to predict the disease-related genes of MS. The whole workflow of the model is shown in Figure 1.

### 2.1. NRL-Based Protein-Protein Interaction Network Embedding

In our method, we use three classical NRL algorithms (node2vec, DeepWalk, and LINE) to capture the global features of the PPI network and represent genes as non-linear feature vectors. The details of the three algorithms are introduced in the next part.

**Figure 2:**
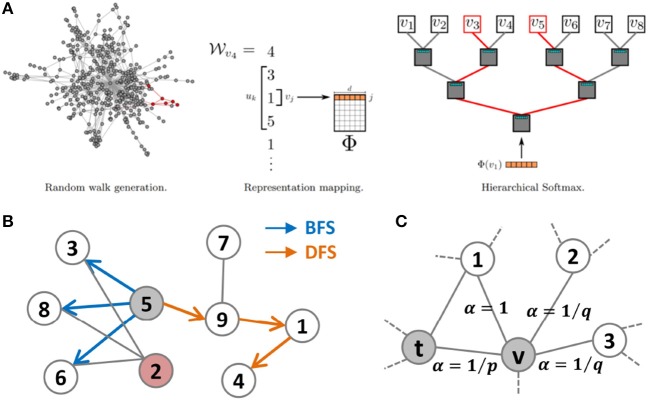
**(A)** Overview of DeepWalk. It consists of three main parts: random walk generation, representation learning, and hierarchical softmax. This figure was extracted from the original paper. **(B)** Two types of search strategies from node 5, BFS and DFS. **(C)** The random walk procedure in node2vec.

DeepWalk (Perozzi et al., 2014) is the first-proposed NRL algorithm. It tries to represent nodes as novel latent feature vectors. It first learns topological information from the network using a random walk algorithm. Then, it can be treated as a natural language process problem. The learned sequence information is inputted into the Skip-Gram model. The aim of the DeepWalk model is to maximize the probability of neighbors of the node *n*_*i*_ in the walk sequence. The objective function can be shown as:

(1) $$\begin{array}{r} max_{\varphi }Pr\left(\left\{n_{i-w},\ldots ,n_{i+w}\right\}\backslash n_{i}|\varphi \left(n_{i}\right)\right)=\overset{i+w}{\underset{j=i-w,j\neq i}{\prod }}Pr\left(n_{j}|\varphi \left(n_{i}\right)\right) \end{array}$$

where *w* is the size of the window and φ(*n*_*i*_) and {*n*_*i*−*w*_, …, *n*_*i*+*w*_} are the current feature representation and neighborhood nodes of *n*_*i*_, respectively. Finally, the DeepWalk algorithm uses hierarchical softmax to generate the low-dimensional representation vectors. The overall overflow can be seen in Figure 2A. node2vec (Grover and Leskovec, 2016) is an extended version of the DeepWalk algorithm. In the process of learning the network topology, node2vec integrates two neighborhood sampling strategies, Breadth-First Search (BFS) and Depth First Search (DFS). These two strategies for capturing topological information are shown in Figure 2B. The node2vec algorithm proposes a novel random walk strategy with two parameters, *p* and *q*. The random walk procedure of node2vec can be seen in Figure 2C. Parameter *p* mainly controls the probability of revisiting a node in the process of random walk, and *q* controls the possibility of capturing “local” or “global” nodes. In particular, if *p* = 1.0 and *q* = 1.0, then the node2vec algorithm can be seen similarly as the DeepWalk method.

LINE (Tang et al., 2015) is designed for large-scale NRL, mainly capturing the first-order and second-order topological information. The idea of second-order information in LINE can be learned from Figure 2B. In this figure, nodes 5 and 2 have the same neighborhood, 3, 8, and 6. Although nodes 2 and 5 are not linked directly, we think that they are similar to each other. The first-order and second-order topological information between two nodes *n*_*i*_ and *n*_*j*_ can be measured as:

(2) $$\begin{array}{r} P_{1}\left(n_{i},n_{j}\right)=\frac{1}{1+exp\left(-u_{i}^{T}u_{j}\right)}\;P_{2}\left(n_{j}|n_{i}\right)=\frac{exp\left({\bar{u}}_{j}^{T}{\bar{u}}_{i}\right)}{\sum_{k}exp\left({\bar{u}}_{k}^{T}{\bar{u}}_{i}\right)} \end{array}$$

where *u*_*i*_ describes the representation of node *n*_*i*_. By optimizing the KL-divergence of these first-order and second-order distributions, we can obtain the final representations of gene nodes.

### 2.2. Extracting Low-Dimensional Feature Vectors

In our NRL-based MS disease-related gene prediction model, we use a stacked autoencoder model to transform high-dimensional non-linear features learned by NRL-based algorithms into low-dimensional feature space. Commonly, many models use Principal Component Analysis (PCA) (Abdi and Williams, 2010) or Independent Component Analysis (ICA) (Hyvärinen and Oja, 2000) to reduce the dimensionality of the feature matrix. However, these methods cannot capture non-linear feature vectors effectively. Also, these linear dimensionality reduction methods would distort the original data structure and cannot keep original features in the low-dimensional feature space. A stacked autoencoder (SAE) model can address these shortcomings.

An autoencoder is an unsupervised model that is widely used in feature extraction and dimensionality reduction. An autoencoder contains two main parts, an encoder and a decoder, and its aim is to minimize the reconstruction error between input and output. The encoded features of the hidden layer are the final low-dimensional output that is used in the downstream tasks. Assuming that the *i*−*th* input node vector is *x*_*i*_, the reconstructed node vector can be described as ${\hat{x}}_{i}=g\left(W^{\prime }\cdot f\left(W\cdot x_{i}+b\right)+b^{\prime }\right)$, where *f* and *g* are activation functions, and Θ = {*W, b, W*′, *b*′} are the parameters to be learned. Then, the loss function of a three-layer autoencoder can be represented as follows:

(3) $$\begin{array}{r} \arg\underset{\theta \in \Theta }{\min}\overset{n}{\underset{i=1}{\sum }}∥{\hat{x}}_{i}-x_{i}{∥}_{2}^{2} \end{array}$$

The stacked autoencoder has been widely used in many areas to extract feature vectors and reduce the dimensionality (Peng et al., 2019b). Thus, we also add a stacked autoencoder model in our framework to improve the performance of predicting MS disease-related genes.

### 2.3. Predicting Disease-Related Genes Based on an SVM Classifier

After obtaining low-dimensional gene feature vectors, we train the SVM algorithm to predict the disease-related genes of MS. This prediction task can be treated as a label classification problem. SVM is applied widely on many classification tasks because of its stability, simplicity, and effectiveness. Here, we also select SVM as the classifier for our model. The disease-related genes of MS are chosen as positive samples, and then we randomly select several unrelated genes as negative samples from the PPI network. The number of negative samples is the same as that of positive samples.

In order to evaluate the performance of the SVM classifier in the task of MS disease-related gene prediction, we randomly select 80% of the dataset as a training dataset and 20% as the test dataset. We choose the standard RBF kernel for the SVM classifier and use the grid search method to select the optimal hyper-parameters.

## 3. Results

### 3.1. Datasets and Baselines

In the experimental part, we mainly use two datasets: the protein-protein interaction network (PPI) and the disease-related genes of MS. The PPI network contains 13,460 nodes and 141,296 edges, which is the same as in the paper (Menche et al., 2015). Candidate genes associated with MS disease were downloaded from the DisGeNet database (https://www.disgenet.org/browser/0/1/1/C0026769) (Pinero et al., 2017). After preprocessing, we can obtain 924 genes that relate to MS disease. In order to evaluate the performance of our proposed method, we compare NRL-based methods with three classical methods, including Random Walk with Restart (RWR) (Li and Patra, 2010), Shortest Path Length (SPL) (Krauthammer et al., 2004) and Euclidean distance (ED) (Díaz-Uriarte and de Andrés, 2006). Random walk with restart is a classical path learning method, which is widely used in biological network analysis to capture the topological structure of the network. Shortest path length and Euclidean distance are both typical path-based disease-related gene prediction methods. We, in this paper, compare NRL-based methods with these path-based methods to validate the superiority of NRL on the task of disease-related gene prediction.

On the task of disease-related gene prediction, we adopt accuracy, F1, area under the ROC curve (AUROC), and area under the PR curve (AUPRC) as the evaluation criterion. All of the experiments adopt five-fold cross-validation. After several experimental validations, the optimal number of dimensions of the PPI network embedding and the final dimensionality of features after running stacked autoencoder are 512 and 64, respectively.

### 3.2. Performance in Predicting Disease-Related Red Genes of MS

In order to validate the performance of NRL-based algorithms on the task of predicting the disease-related genes of MS, we compare our model with three classical methods: random walk with restart, shortest path length, and Euclidean distance. The experimental results of the NRL-based methods and baselines are shown in Table 1. The node2vec-based and DeepWalk-based methods are obviously superior to the other algorithms. For node2vec, the values of accuracy and AUROC reach 0.7011 and 0.7647, respectively, much higher than the three classical methods. The performance of DeepWalk is similar to that of node2vec, and the AUPRC value of DeepWalk is the highest among the six algorithms. However, the performance of LINE is not as good as the other two NRL-based methods. LINE mainly considers the first-order and second-order information of the network topology in the process of embedding. The PPI network is very sparse and many isolated nodes exist, which may lead to the poor performance of LINE. Overall, the NRL-based methods contribute to improving the performance of MS disease-related gene prediction.

**Table 1:** The experimental results of NRL-based methods and other baselines.

	**Abc**	**F1**	**AUROC**	**AUPRC**
ED	0.6032 (0.0165)	0.5933 (0.0204)	0.6439 (0.0163)	0.6356 (0.0216)
SPL	0.6136 (0.0296)	0.6033 (0.0198)	0.6703 (0.0205)	0.6531 (0.0208)
RWR	0.5312 (0.0113)	0.5203 (0.0305)	0.5431 (0.0195)	0.5321 (0.0233)
LINE-SAE-SVM	0.5527 (0.0102)	0.5403 (0.0218)	0.5838 (0.0106)	0.5716 (0.0198)
node2vec-SAE-SVM	**0.7011 (0.0212)**	**0.6944 (0.0138)**	**0.7647 (0.0186)**	0.7472 (0.0283)
DeepWalk-SAE-SVM	0.6941 (0.0288)	0.6914 (0.0315)	0.7554 (0.0204)	**0.7478 (0.0243)**

*The bold values indicate the best performance*.

### 3.3. Effects of Different Parameters on Disease-Related Gene Prediction

**Figure 3:**
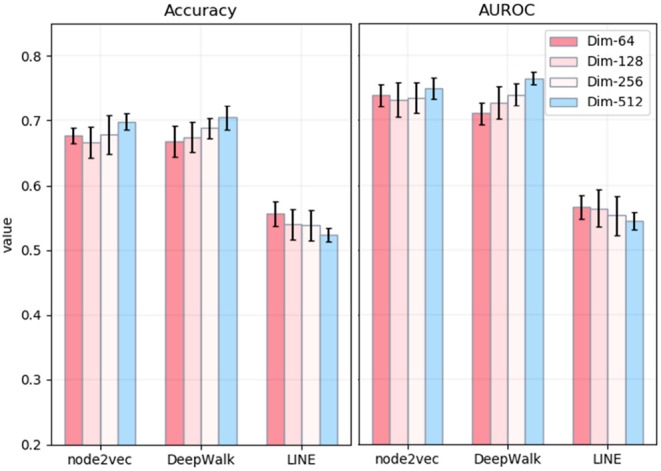
Accuracy and AUPRC values of three network representation learning algorithms with four different numbers of dimensions. The x-axis represents three different methods. The y-axis represents the values of Accuracy **(left)** and AUROC **(right)**.

The whole process of the NRL-based methods consists of three main parts: capturing the topological information of the PPI network, extracting low-dimensional features, and predicting disease-related genes based on the SVM classifier. Among different parameters, the most influential is the number of dimensions of embedding. Thus, we mainly explore the effects of the number of embedding dimensions on the task of disease-related gene prediction. In detail, we run three NRL algorithms with four different numbers of dimensions, namely 64, 128, 256, and 512. The experimental results are shown in Figure 3. In general, the values of accuracy and AUROC are stable, and the number of embedding dimensions has less impact on the experimental results in predicting the disease-related genes of MS. For node2vec, the values of accuracy and AUROC are around 0.67 and 0.73, respectively, in the case of the four different dimensionalities.

**Figure 4:**
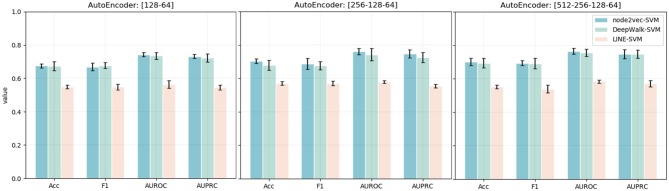
Accuracy, F1, AUPRC, and AUPRC values of three network representation learning algorithms with four different numbers of dimensions and different autoencoder structures. The x-axis represents four different evaluation metrics. The y-axis represents the value of the evaluation metric.

Except for the dimensionality of network embedding, we also consider the effects of the stacked autoencoder. Here, we also embed the PPI network with four different numbers of dimensions. We, then, implement the stacked autoencoder to transform high-dimensional features into low-dimensional space. The final number of dimensions through the stacked autoencoder is 64. The experimental results are shown in Figure 4. Comparing the experimental results with the model without an autoencoder, we can clearly see the effects of the autoencoder on extracting low-dimensional features. Besides, with the increase in the number of autoencoder layers, the model shows better performance in the task of predicting MS disease-related genes. Thus, we adopt five layers [512-256-128-64] as our model's stacked autoencoder structure. In the third part, an SVM classifier is used in our model to predict disease-related genes. This step is flexible: we can train other classifiers to finish prediction tasks. Here, we also train Logistic Regression and Random Forest classifiers to predict the disease-related genes of MS. The detailed experimental results are shown in Table 2.

**Table 2:** The experimental results of NRL-based methods with different classifiers.

		**Acc**	**F1**	**AUROC**	**AUPRC**
Logistic	LINE	0.5272(0.0131)	0.5172(0.0125)	0.5596(0.0138)	0.5391(0.0248)
Regression	node2vec	0.6483(0.0163)	0.6483(0.0163)	0.6899(0.0236)	0.6409(0.0208)
	DeepWalk	0.5793(0.0250)	0.5793(0.0150)	0.6658(0.0216)	0.6153(0.0200)
Random	LINE	0.6176(0.0188)	0.6276(0.0188)	0.6208(0.0216)	0.6057(0.0263)
Forest	node2vec	0.7172(0.0117)	0.7012(0.0217)	0.7400(0.0126)	0.7191(0.0203)
	DeepWalk	0.6959(0.0215)	0.6759(0.0163)	0.7336(0.0185)	0.7008(0.0202)

**Figure 5:**
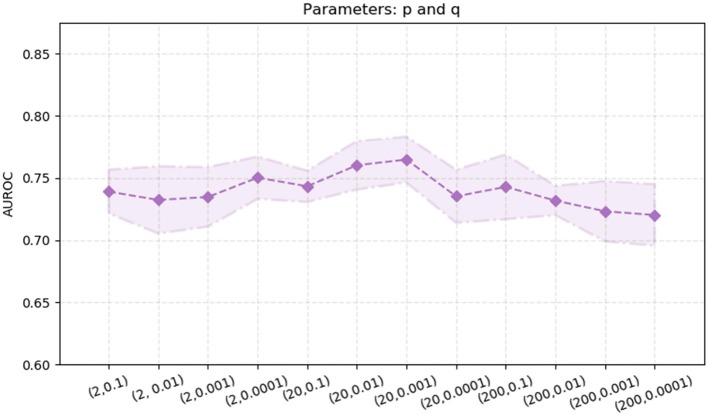
AUROC with different parameter combinations of *p* and *q* in the node2vec algorithm. The x-axis represents different parameter combinations. The y-axis represents the value of AUROC.

node2vec performs better than the other two algorithms, DeepWalk and LINE. Thus, we also explore the effects of the two parameters in the node2vec algorithm, *p* and *q*. We randomly select parameters *p* ∈ {2.0, 20.0, 200} and *q* ∈ {0.1, 0.01, 0.001, 0.0001}. The experimental results are shown in Figure 5. The AUROC values are fluctuating within a certain range [0.72, 0.77]. When *p* = 20 and *q* = 0.01, the AUROC value of the node2vec algorithm achieve its maximum (0.7647).

## 4. Conclusion

Identifying the disease-related genes of MS effectively is essential for the treatment and diagnosis of MS. In this paper, we introduce NRL methods into the task of identifying disease-related genes and propose a novel NRL-based framework to predict the disease-related genes of MS. The NRL-based algorithms consist of three main components: capturing the global topological structure of the PPI, encoding non-linear representation vectors into low-dimensional feature space using a stacked autoencoder, and training a SVM classifier to predict disease-related genes. We compare our proposed method with three classical algorithms. The experimental results show the superior performance of the NRL-based algorithms. Moreover, the proposed NRL-based algorithms are scalable and robust enough to be applied to many other tasks of disease-related gene prediction.

## Data Availability Statement

Publicly available datasets were analyzed in this study. This data can be found here: https://www.disgenet.org/browser/0/1/1/C0026769.

## Author Contributions

HLiu formulated the study concept and designed the study. HX, JG, and HLi performed research and implemented the algorithm. HX and HLi wrote the paper. QW, ZB, and XL designed the experiments and wrote the paper. All authors read and approved the final manuscript.

## Conflict of Interest

The authors declare that the research was conducted in the absence of any commercial or financial relationships that could be construed as a potential conflict of interest.
